# Left ventricular mass is underestimated in overweight children because of incorrect body size variable chosen for normalization

**DOI:** 10.1371/journal.pone.0217637

**Published:** 2019-05-29

**Authors:** Hubert Krysztofiak, Marcel Młyńczak, Łukasz A. Małek, Andrzej Folga, Wojciech Braksator

**Affiliations:** 1 Mossakowski Medical Research Centre, Polish Academy of Sciences, Warsaw, Poland; 2 National Centre for Sports Medicine, Warsaw, Poland; 3 Warsaw University of Technology, Faculty of Mechatronics, Institute of Metrology and Biomedical Engineering, Warsaw Poland; 4 Faculty of Rehabilitation, Józef Piłsudski University of Physical Education, Warsaw, Poland; 5 Department of Sports Cardiology and Noninvasive Cardiovascular Imaging, 2nd Medical Faculty, Medical University of Warsaw, Warsaw, Poland; University of Oslo, NORWAY

## Abstract

**Background:**

Left ventricular mass normalization for body size is recommended, but a question remains: what is the best body size variable for this normalization—body surface area, height or lean body mass computed based on a predictive equation? Since body surface area and computed lean body mass are derivatives of body mass, normalizing for them may result in underestimation of left ventricular mass in overweight children. The aim of this study is to indicate which of the body size variables normalize left ventricular mass without underestimating it in overweight children.

**Methods:**

Left ventricular mass assessed by echocardiography, height and body mass were collected for 464 healthy boys, 5–18 years old. Lean body mass and body surface area were calculated. Left ventricular mass z-scores computed based on reference data, developed for height, body surface area and lean body mass, were compared between overweight and non-overweight children. The next step was a comparison of paired samples of expected left ventricular mass, estimated for each normalizing variable based on two allometric equations—the first developed for overweight children, the second for children of normal body mass.

**Results:**

The mean of left ventricular mass z-scores is higher in overweight children compared to non-overweight children for normative data based on height (0.36 vs. 0.00) and lower for normative data based on body surface area (-0.64 vs. 0.00). Left ventricular mass estimated normalizing for height, based on the equation for overweight children, is higher in overweight children (128.12 vs. 118.40); however, masses estimated normalizing for body surface area and lean body mass, based on equations for overweight children, are lower in overweight children (109.71 vs. 122.08 and 118.46 vs. 120.56, respectively).

**Conclusion:**

Normalization for body surface area and for computed lean body mass, but not for height, underestimates left ventricular mass in overweight children.

## Introduction

In children, as the body grows with age, the size of the heart also increases. Body size is a major determinant of cardiac size. Among different body size variables, lean body mass (LBM) seems to be the strongest predictor of the heart chamber size and wall thickness [1–3]. However, fat mass of the body, though not as strong a determinant of cardiac size as LBM, also has biological and clinical significance [1,2]. Obese children have increased left ventricular mass (LVM) compared to their lean peers, independently of other pathological determinants such as, e.g., hypertension [4,5], and overweight and obesity are common problems in children and adolescents [6]. The problem of excess body mass is also observed in young athletes [7,8], in whom regular physical activity is a strong determinant of left ventricular hypertrophy (LVH). In overweight or obese athletes, an effect of adiposity may be partially responsible for LVH [9].

To effectively evaluate cardiac size, proper normalization is necessary, especially in growing children and adolescents, because of high variability in height and body mass, even among similar aged children. As cardiac size depends on body size, it is generally accepted that normalization for body size is optimal for cardiac size scaling. However, one important question remains open: which independent variable, representing body size, is the best to use?

Body surface area (BSA) seems to be the most-used scaling variable [10]. However, BSA calculation takes into account both the lean and fat components of body mass, so normalization for BSA may hide the influence of excess body mass, adiposity or musculature for example, on cardiac size, producing underestimated results [4,11,12]. This effect is referred to as a bias related to body mass index (BMI) or BMI bias [11].

Height is another body size variable used in normalization of cardiac size. Height raised to the power of 2.7 is the most often used and is recommended by guidelines for LVM scaling [13]. Height seems to be free of the bias related to BMI in the context of cardiac size normalization [4,5,14]. However, in one 2013 study, it was suggested that when the height is used as a scaling variable, the LVM is overestimated in comparison to the results of scaling for BSA or LBM in overweight children [15].

Lean body mass (LBM) has been recommended for left ventricular mass normalization [15–17]. It is potentially the best body size variable for cardiac size normalization because it is the strongest determinant of cardiac size. However, LBM is not easy to measure in daily clinical practice, so equations to compute LBM, based on estimated models [18], have gained popularity [15,17]. Since the predictive equations to compute LBM use body mass and body mass index as explanatory variables, a doubt arises about their effectiveness in the context of BMI bias.

The aim of this study is to indicate, in an independent manner, which of the three body size variables used for cardiac size normalization, i.e., height, BSA or computed LBM, provide normalization without underestimation of the left ventricular mass in overweight children which one is free of the bias related to BMI.

## Materials and methods

### The study group

This retrospective study was carried out on echocardiography data from child and adolescent male athletes examined during periodic preparticipation physical evaluation (PPE) at the National Center for Sports Medicine.

The study group consisted of 464 healthy white boys, 5–18 years old, engaged in regular sport activities. All the study participants had undergone transthoracic echocardiography as a part of periodic PPE due to innocent heart murmurs or suspicion of abnormal electrocardiographic findings. Children in whom echocardiography revealed significant acquired or congenital heart diseases, affecting normal heart size and hemodynamics, were excluded.

### Anthropometric measures

Height and body mass were measured during the PPE examination. Body mass index was calculated and expressed as a z-score in relation to age. The BMI z-scores were computed based on our own analysis of the study group. The LMS method, originally introduced by Cole [19], was chosen to create growth curves and calculate the z-scores. The same method was used to develop the 2007 World Health Organization reference data for BMI [20]. Body surface area was calculated according to the Haycock formula [21], which is recommended for the pediatric population [22]:

$$BSA[m^{2}]=0.024265\cdot {Height[cm]}^{0.3964}\cdot {Body\;mass[kg]}^{0.5378}$$

Lean body mass was computed according to a formula introduced by Foster BJ et al. [18]:

$$LBM[kg]=exp\{-2.8900+0.8064∙ln(Height[cm])+0.5674∙ln(Body\;Mass[kg])+0.0000185∙{\left(Body\;Mass[kg]\right)}^{2}-0.0153∙{\left(BMIz_{score}[kg]\right)}^{2}+0.0132∙(age[years])\}$$

### Echocardiography

Echocardiograms were performed by two experienced sonographers using a commercially available ultrasound scanner (Toshiba Aplio 400, Toshiba Medical Systems Europe, Zoetermeer, the Netherlands), according to recent guidelines. All measurements were taken in 2-dimensional parasternal long axis view (PLAX) and included basic linear cardiac dimensions necessary for LVM computing: left ventricular (LV) cavity dimension in end-diastole, interventricular septum diameter at end diastole (IVSd), left ventricular posterior wall diameter at end diastole (PWD). All measurements were taken from inner edge to inner edge and reported to within 1 mm. Subjects exhibiting ambiguous results of any measurement were excluded from the initial study group. Left ventricular mass was computed according to the well-known formula originally introduced by Devereux RB et al [23]:

$$LVM=0.8\{1.04[LV\;cavity\;dimension+PWD+{IVSd)}^{3}-{\left(LV\;cavity\;dimension\right)}^{3}\}+0.6$$

### Ethical considerations

The study, including the consent procedure, was approved by the Ethics Committee of the Medical University of Warsaw (approval AKBE/75/17). As the study was retrospective neither written nor verbal consent was obtained for this particular study, but each subject had signed the informed consent form for the routine medical monitoring, including a statement of agreement to the use of the results for scientific purposes.

### A preliminary exploration of BMI bias in normalized LVM values

Left ventricular mass is higher in overweight and obese children compared to children of normal body mass [1,14,24,25,26,27,28]. To test the reliability of the normalizing variable, LVM z-score values of overweight children were compared with LVM z-score values of children with normal body mass using successively three different LVM reference data normalized for height, BSA and computed LBM, respectively.

The LVM reference data were developed based on our study group. Three sets of centile curves were generated from the distributions of raw values of LVM relative to height, BSA and computed LBM. The same methodology like in recent studies of Foster BJ et al. [15,17] was used. The LMS method [19] was applied to generate expected LVM mean (M), coefficient of variation (S) and skewness (L) for each level of height, BSA or computed LBM.

For each subject of the study group three LVM z-score values were calculated based on the three reference data sets developed for height, BSA and computed LBM, respectively. The z-scores were calculated from the L, M and S values corresponding to the child’s actual height, BSA and computed LBM according to the equation:

$$z-score=\frac{\left[{\left(\frac{actual\;LVM}{M}\right)}^{L}-1\right]}{L∙S}$$

Next, the study group was divided into two subgroups based on BMI z-score. To the first subgroup (NORM) only non-overweight and non-obese children, with BMI z-score <1, were qualified. To the second subgroup (OVER), overweight and obese children, with BMI z-score>1, were qualified. The T test for independent samples was used to compare the respective mean LVM z-score values of overweight children and children with normal body mass.

### A method for analysis of bias related to BMI introduced by the normalizing variable

We developed a method for analyzing the BMI-related bias introduced by the normalizing variable. The method is based on an assumption that LVM in overweight subjects is higher than LVM in subjects with normal body mass. It starts with the development of two allometric equations to predict LVM based on the body size variable being tested as a normalizing variable. The first equation is developed using data from all overweight and obese subjects in the study group. The second is developed using data from all subjects with normal body mass.

Based on these equations, two expected LVM values are computed for each subject in the study group. The first value represents LVM expected for overweight subjects, and the second value represents LVM expected for subjects with normal body mass. As a result, two paired samples of the expected LVM values are generated. The basis of the method is to compare these paired samples. An extension to the method is a visual presentation of these two samples on a single scatter graph against the BMI z-score and a comparison of the regression coefficients of the two lines fitted to the expected LVM values in the respective samples. For demonstration, one might show on a single graph the LVM-for-body size curves defined by the allometric equations.

In the case where the LVM normalization uses a body size variable which is free of BMI bias, the mean of expected LVM computed based on the allometric equation developed for overweight subjects should be higher than the mean of expected LVM computed based on the equation developed for subjects with normal body mass. In principle, in each pair, the expected LVM value that is representative for overweight subjects should be higher than the representative value for subjects with normal body mass.

Consequently, on the combined scatter graph of the two expected LVM values against BMI z-score, a regression line fitted to the expected LVM values computed based on the equation developed for overweight subjects should be set above the corresponding line fitted to the expected LVM values computed based on the equation for subjects with normal body mass, across the entire range of BMI z-scores.

This has to be confirmed by a comparison of these lines. That can be done by analyzing the regression coefficients of a line fitted to the differences between paired expected LVM values against BMI z-score. The y-intercept of the regression line fitted to the differences should be significant, and the slope non-significant.

### The BMI bias analysis method in practice

Bivariate allometric equations were fitted by nonlinear least squares to determine the expected LVM from each of height, BSA and computed LBM. A method introduced by Marquardt was used [29]. The equations were developed in parallel for separate data, from the OVER subgroup and from the NORM subgroup. Thus, for each of the three body size variables two separate, subgroup-specific, equations were developed; a total of three pairs of allometric equations for each subject. These equations have the general form *LVM* = *a*(*body size*)^*b*^, where *a* and *b* are the coefficient and exponent of the allometric equation, respectively.

According to these equations, three pairs of expected LVM values were calculated for each child in the study group. The paired expected LVM values were then plotted against the BMI z-score–a separate graph for each explanatory variable. Regression lines for the expected LVM values corresponding to the OVER and NORM equations were fitted on each scatter graph and the linear regression coefficients were estimated.

In order to compare the coefficients of the regression lines, differences between the expected LVM values in each pair were calculated. Next, linear regression coefficients for the relationship between each set of these differences and BMI z-score were tested.

All calculations were conducted using R (version 3.4.2, “Short Summer”; R Foundation, Vienna, Austria, http://www.r-project.org), along with external packages, especially the gamlss package (version 5.0) which contains a function to fit LMS curves. For all statistical tests, a significance level of α = 0.05 was applied.

## Results

### Subjects characteristics

Based on BMI z-score, 394 boys were qualified to the NORM subgroup and 70 were qualified to the OVER. The LVM reference data for exploratory analysis were developed on the NORM subgroup. Detailed characteristics of the study group and the subgroups are presented in Table 1.

**Table 1:** Study group characteristics.

	Entire group	NORM subgroup	OVER subgroup	p
Number of subjects	464	394	70	
Age (years)	12.17 (3.22)	12.26 (3.26)	11.70 (2.96)	ns
Height (m)	1.58 (0.20)	1.58 (0.20)	1.59 (0.18)	ns
Body mass (kg)	49.04 (17.89)	47.21 (17.15)	59.33 (18.61)	< 0.001
computed LBM (kg)	37.05 (13.80)	36.42 (13.62)	40.59 (14.33)	< 0.05
BSA (m^2^)	1.45 (0.35)	1.42 (0.35)	1.61 (0.35)	< 0.001
BMI z-score	0.01 (0.99)	-0.27 (0.78)	1.57 (0.43)	< 0.001

Data are expressed as mean (standard deviation); BSA, body surface area, was calculated based on the Haycock formula; computed LBM, computed lean body mass, was calculated according to a formula introduced by Foster BJ et al. [18].

### Exploratory analysis of BMI bias in normalized LVM values

Three sets of reference LVM data, generated for height, BSA and computed LBM, are presented as the L, M and S numbers in supplemental text files (S2 File, S3 File, S4 File).

The comparison of the respective mean LVM z-score values of overweight children and children with normal body mass is presented in Table 2. Only for the LVM z-scores computed upon the height-based reference data, the normalized LVM is significantly higher in overweight children compared to children of normal body mass. The reverse picture is seen for the LVM z-scores computed upon the reference data based on BSA—the LVM z-scores are significantly higher in the children of normal body mass compared to the overweight children. Similarly for the LVM z-scores calculated upon the reference data based on computed LBM, the normalized LVM is lower in the overweight children compared to the children of normal body mass, but the difference is not statistically significant.

**Table 2:** The mean LVM z-scores of overweight children (OVER) and children with normal body mass (NORM) depending on the LVM reference data used.

	OVER	NORM	p
LVM z-scores by			
Height	0.36 (0.94)	0.00 (1.00)	< 0.01
BSA	-0.64 (1.02)	0.00 (1.00)	< 0.001
computed LBM	-0.11 (1.06)	0.00 (1.06)	ns

Data are expressed as mean (standard deviation); BSA, body surface area; computed LBM, computed lean body mass.

### Analysis of BMI bias introduced by normalizing variable

**Fig 1:**
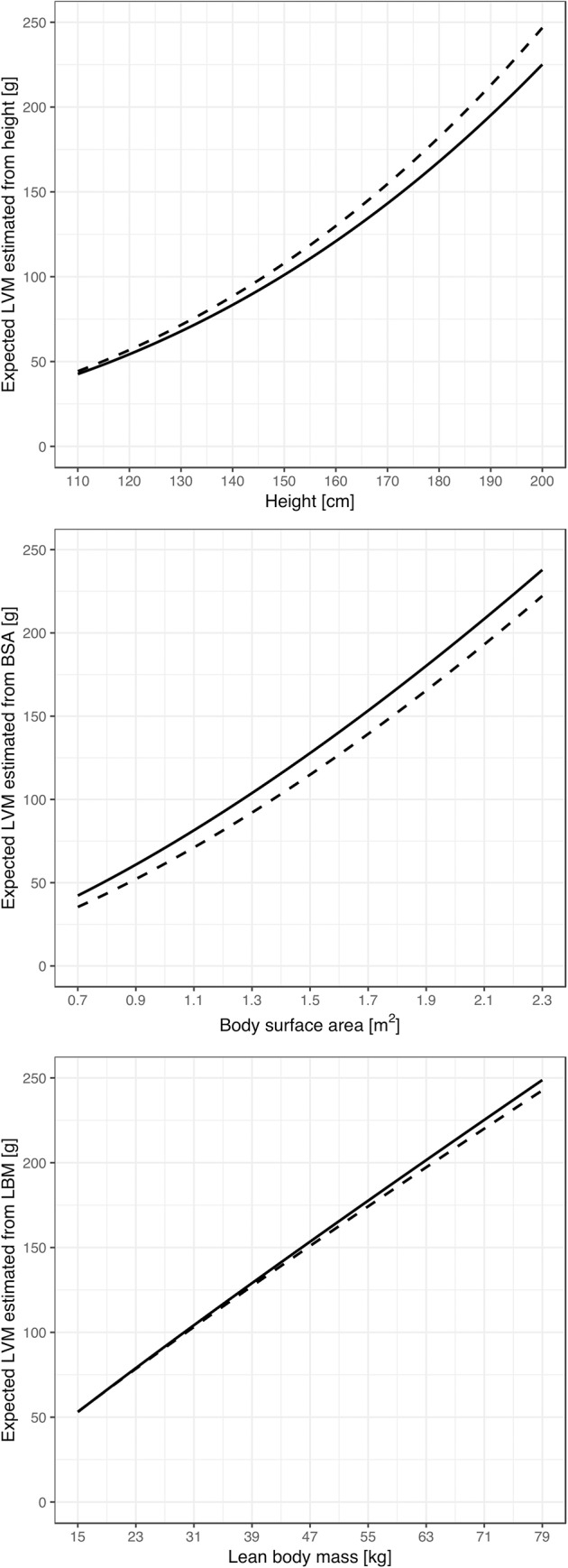
The pairs of the LVM-for-body size curves defined by the allometric equations, corresponding to the OVER (dashed line) and NORM (solid line) groups. The upper graph presents the LVM-for-height curves, the middle LVM-for-BSA, and the lower graph the LVM-for-computed LBM curves.

Coefficients and exponents of the LVM predictive allometric equations developed for each of the explanatory variable, i.e., height, BSA and computed LBM and for each subgroup, are presented in supplemental S1 Table. The pairs of corresponding LVM-for-body size curves defined by the allometric equations are demonstrated in Fig 1.

Only for height the expected LVM value representative for overweight subjects is higher than the representative value for subjects with normal body mass. Minimum individual difference is 1.80, and maximum individual difference is 20.40. For BSA, both the minimum and maximum differences are negative (-7.30 and -15.65, respectively). For LBM, the range of differences is from -5.84 (negative) to 0.26 (positive).

**Fig 2:**
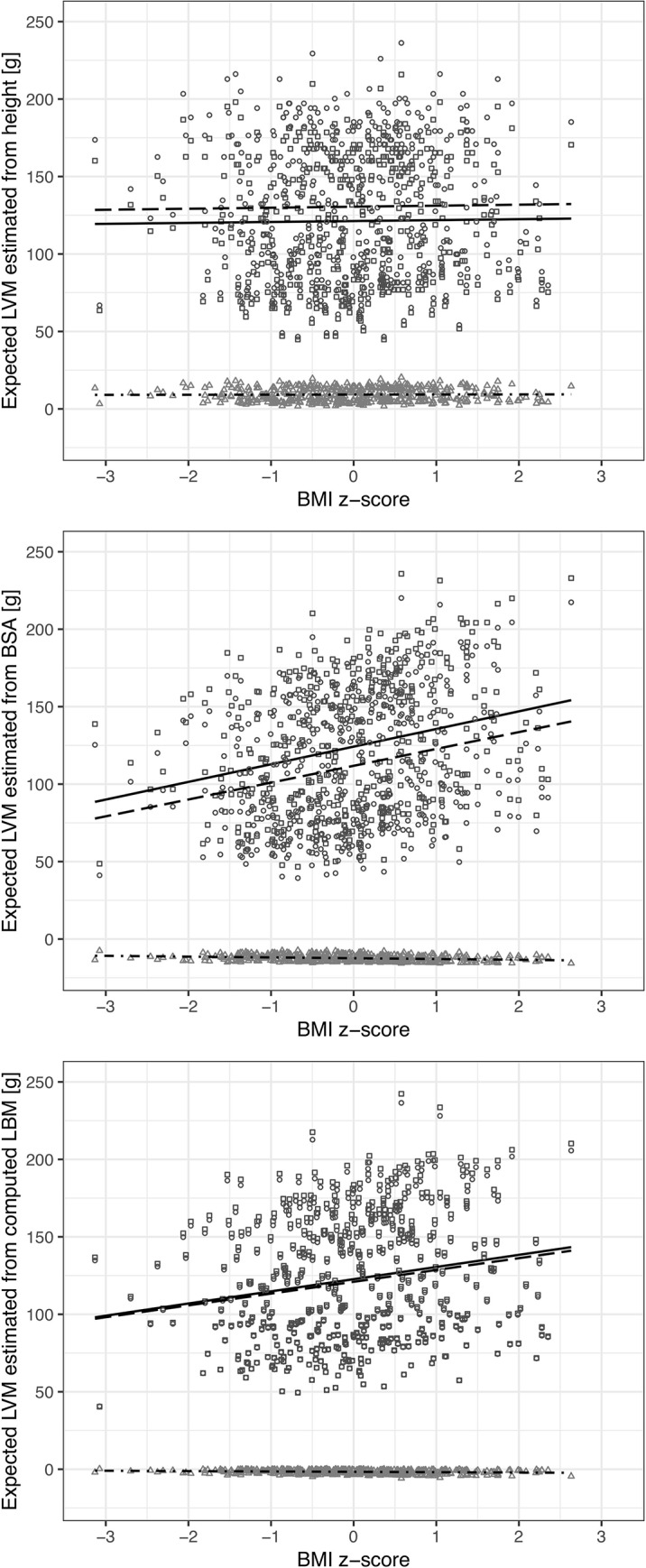
Scatter graphs of paired LVM values corresponding to the OVER (square) and the NORM (circle) equations and of differences between these paired values (triangle) plotted against BMI z-score. Respective graphs show data which correspond to different explanatory variables, i.e., height, BSA and computed LBM. On each graph regression lines for the expected values and the differences are fitted–the solid line corresponds to the data computed based on the NORM equation, the dashed line corresponds to the data computed based on the OVER equation, and the dot-dash line corresponds to the differences.

Pairs of the expected LVM values computed based on the OVER and the NORM equations, respectively, are plotted against BMI z-score on separate scatter graphs corresponding to each explanatory variable (Fig 2). Additionally, differences between the paired expected LVM values are plotted on the graphs. Regression lines for these paired expected LVM values and for the differences are drawn on each scatter graph. The coefficients of the regression lines, slopes and y-intercepts, are presented in Table 3.

**Table 3:** Comparison of linear regression coefficients for relationships between expected LVM values and BMI z-score. For each explanatory variable the expected LVM values are calculated twice. First, based on a predictive equation developed for the OVER subgroup, next on a predictive equation developed for the NORM subgroup. In order to compare these linear regression coefficients differences between the paired expected LVM values were calculated and linear regression coefficients for the relationship between the differences and BMI z-score were tested.

	Slope	p	Intercept	p
**LVM estimated from height**				
equation for OVER	2.08	ns	129.93	< 0.001
equation for NORM	1.88	ns	120.74	< 0.001
Difference	0.20	ns	9,19	< 0.001
**LVM estimated from BSA**				
equation for OVER	12.20	< 0.001	111.63	< 0.001
equation for NORM	12.78	< 0.001	123.97	< 0.001
Difference	-0.58	< 0.001	-12.34	< 0.001
**LVM estimated from computed LBM**				
equation for OVER	8.70	< 0.001	120.71	< 0.001
equation for NORM	8.97	< 0.001	122.37	< 0.001
Difference	-0.27	< 0.001	-1.66	< 0.001

The only lines set in proper order are the lines for height. The line representing the expected LVM computed according to the equation developed for overweight children is above the line fitted to the expected LVM values predicted based on the equation for children of normal body mass. The intercepts of the lines are significantly different because the intercept of the differences’ regression line is statistically significant. These intercepts are equal to the means of the respective expected LVM values. Thus, the mean of the expected LVM values predicted based on the OVER equation is higher than the mean of the expected LVM values predicted based on the NORM equation. The slopes of both the lines are non-significant, and the lines are parallel because the slope of the line for the differences is also non-significant.

In the cases of BSA and computed LBM, the line representing the expected LVM computed according to the equation developed for overweight children is set below the line fitted for the expected values predicted based on the equation developed for children with normal body mass. The intercepts of the lines are significantly different as confirmed by the statistically significant intercept of the line for the differences. Thus, the mean of the expected LVM values computed based on the OVER equation is lower than the mean of the expected values computed based on the NORM equation. The slopes of all the lines for expected LVM values are significant, and slopes of corresponding lines are significantly different as confirmed by the statistically significant slopes of the respective lines for the differences.

## Discussion

The results of this study show that, for normalization of left ventricular mass for body size, among commonly used scaling variables, like BSA, computed LBM and height, only height allows for reliable evaluation of LVM in children and adolescents. Left ventricular mass normalized for height is not underestimated in overweight and obese children. This is because height as a scaling variable is free of the bias related to body mass index. In turn, LVM normalization for BSA gives misleading results, because normalized LVM is lower in overweight children compared to their lean peers. A similar effect is observed for LVM normalized for computed LBM.

### Relation between LVM and body mass

There is an agreement that excessive fat has a negative impact on the heart resulting in hypertrophy and changes of LV geometry [1,14,24,25,26,27,28]. Cardiac hypertrophy is recognized as a predictor of cardiovascular disease morbidity and mortality in adults [30] and also as a concern in children and adolescents [31]. Therefore, it is important to reliably diagnose LV hypertrophy; it is important to find optimal scaling variable for LVM, especially in growing children and adolescents, due to high variability in height and body mass in similar aged children.

Left ventricular mass normalization for body size is physiologically justified and widely accepted. However, LVM normalization for body mass or its derivatives like BSA, may abate or even eliminate influence of excess body mass producing false normal results [4,5,11,12,32]. The problem of bias related to BMI or more generally, bias related to body mass in cardiac size scaling, has been raised many times for more than twenty years [4,5,33]. However, BSA is still commonly used as a cardiac size normalizing variable [34,35,36], although any scaling approach that eliminates the positive residual relation between normalized LVM and BMI or body mass is biased. This is discussed comprehensively by Woodiwiss AJ and Norton GR [37] in their state of the art review article.

### Lean body mass is the best LVM scaling variable, but impractical

Normalization for LBM seems to be ideal, since LBM is recognized as the strongest determinant of LVM. Research in athletes shows that changes in LVM related to training can be explained by parallel changes in LBM [38,39,40]. The expected effect of scaling LVM for LBM should be "complete normalization": in healthy children of normal body mass, the normalized LVM would be constant across changes in body size. Specific normative data for athletes would not be necessary. Certainly, the influence of body fat on LVM would be apparent because LBM is essentially free of the bias related to BMI.

However, LBM is impractical in everyday clinical practice because its measurement requires advanced methods like dual x-ray absorptiometry, air displacement plethysmography, computed tomography or magnetic resonance imaging. Therefore, the LBM predictive equations based on body size variables, including body mass and their derivatives, are used. Unfortunately, the recently introduced computed lean body mass [15,18] is not free of BMI bias. It is true that there was an attempt to examine the bias related to adiposity and to prove that the equation based LBM is free of this bias [15]. However, it seems that the final result, favoring the computed LBM and even BSA against body height, is fraught with a logical error, because the analysis is based on an assumption that computed LBM is the reference variable for cardiac size scaling; without proving that it is free of BMI bias. The results of our study show that the reliability of computed LBM in the context of BMI bias is weak and similar to that of BSA.

### Height should be the standard variable for cardiac size normalization

The exploratory analysis and the method for analysis of BMI bias we used indicated inconsistency in evaluation of LVM when body size variables being derivatives of body mass, like BSA and computed LBM, were applied as normalizing variables for cardiac size normalization. Normalized LVM in children with higher relative body mass, as expressed by BMI z-score, was lower compared to children with moderate BMI. Only LVM normalized for height showed a normal pattern of higher normalized LVM in children with higher BMI z-scores. This indicates that height should be the first choice body size variable for cardiac size normalization. In addition, "height in humans is a classic quantitative trait, easy to measure with precision" [41], resistant to environmental influences, in contrast to body mass, which may show large intra-individual variability in response to different caloric intake, regardless of changes associated with body growth [41, 42]. Of course, scaling of LVM for height is not the "complete normalization." In healthy children of normal body mass, LVM normalized for height has a significantly positive relation with body mass and lean body mass. And though specific normative data for athletes are needed, the effect of LBM on the heart can be evaluated. The same goes for the effect of fat mass on the heart, since height is for sure free of bias related to BMI.

### Study limitations

Our study has some limitations. We introduced a method for analysis of BMI bias, based on an assumption that overweight and obese children have increased left ventricular mass, compared to their lean peers. Although this is confirmed in many studies [1,14,24,25,26,27,28] and there is no study presenting the opposite position, one may argue the lack of gold standard methodology of LVM normalization prohibits this assumption from being considered as an axiom. However, from a biological perspective there is no logical explanation for lower LVM in healthy subjects with higher BMI compared to those with lower BMI, and such a picture was shown in our study in the case of BSA used as a scaling variable.

Our research focused on normalizing variables, not on normalization methods. In exploratory analysis the centile curve construction procedure based on LMS methodology [17,19] was used, and in the method of BMI bias analysis an allometric methodology was applied. It cannot be precluded that the application of a more advanced method of LVM normalization may limit the BMI bias, but because the bias is introduced by the normalizing variable, regardless of which procedure is used, the method will still be burdened with some degree of error of the normalizing variable.

In this research, we decided to use BMI z-score reference data developed specifically for our study group. This may be considered a limitation because the LBM formula validated by Foster BJ et al. is based on National Center for Health Statistics reference data [18]. However, since BMI is influenced almost equally by both the lean body mass and the fat mass, in our opinion, applying BMI reference data from the general population to this athletic group could cause an overestimation of the z-score values, exaggerating the number of subjects classified as overweight with excess body fat. It should be noted that the BMI z-score term in this equation has a negative coefficient. This is because its role is to correct the computed LMB by subtracting the fat mass. In an athlete with high muscle mass, an overestimated BMI z-score would produce an underestimated LBM value.

We used the same study group for development of reference LVM data and subsequent comparison of the normalized LVM values. The same data were used for the development of allometric equations for expected LVM predicted from height, BSA and computed LBM and calculation of the expected LVM values. The composition of the subgroups of overweight children and children of normal body mass resulted from simple division of the study group based on BMI z-score. Therefore, the OVER subgroup consisted of substantially fewer children. This could affect computation of the reference and the predictive LVM data. However, this was a retrospective study and the simple exploratory analysis showed clear inconsistency in LVM values normalized for BSA or computed LBM when comparing overweight and non-overweight children. This was confirmed when the method of analysis of bias related to BMI was used. It seems that if we had had separate group of overweight children, we would have obtained even more distinct results.

The study group consisted only of male child and adolescent athletes and the specificity of this group and lack of female subjects can be considered limitations. Athletes are our group of interest. We consider this group especially valuable for the analysis of BMI bias in LVM normalization. This is because for this group it is more evident that the BMI bias is related not only to adiposity, but also to LBM. Since the study is generally an analysis of a bias related to body mass, one may consider this specificity both a limitation and an advantage.

We decided to limit this analysis to male subjects, knowing that after the inclusion of female subjects to the study, separate groups and parallel tests will be necessary, as in the case of the development of normative data for LVM. However, this is not a presentation of LVM reference values. The idea was to construct a statistical method to check the reliability of explanatory variables used for LVM normalization and to use the method to indicate the most reliable, BMI bias-free, body size variable for LVM normalization, without prior assumption that one of the variables is a reference variable. Parallel testing of the male group and female group would be the double-check to the method. Although the absence of female subjects can be considered a limitation, we are convinced that this is not a critical drawback.

## Conclusions

We conducted an analysis, which has shown that when BSA or equation-based LBM are used for normalization of LVM, the normalized LVM is underestimated in overweight children. This is because these body size variables are derivatives of body mass and introduce an error to the normalized LVM, referred to as bias related to BMI. This analysis has also indicated that only height-based normalization of LVM is free of the BMI bias. Left ventricular mass normalized for height shows a correct pattern of higher normalized LVM in overweight children compared to children of normal body mass. A key practical message from this study is that height should be a body size variable of the first choice for cardiac size normalization.

## Supporting information

S1 File Study dataset. (CSV)

S2 File Reference LVM data generated for height. (CSV)

S3 File Reference LVM data generated for BSA. (CSV)

S4 File Reference LVM data generated for computed LBM. (CSV)

S1 Table The coefficients and exponents of the allometric equations for calculating the expected LVM from height, BSA and computed LBM respectively, for the OVER and NORM subgroups. These allometric equations have the general form *LVM* = *a*(*body size*)^*b*^, where a and b are the coefficient and exponent of the allometric equation, respectively.
(DOCX)
