# Supplementary material for: Left ventricular mass is underestimated in overweight children because of incorrect body size variable chosen for normalization
Source: PLoS One. 2019 May 29;14(5):e0217637. doi: 10.1371/journal.pone.0217637 (PMC6541472; doi:10.1371/journal.pone.0217637)
Supplement: S1 Table — These allometric equations have the general form LVM = a(body size)b, where a and b are the coefficient and exponent of the allometric equation, respectively. (DOCX) [file pone.0217637.s005.docx]

**S1 Table. The coefficients and exponents of the allometric equations for calculating the expected LVM from height, BSA and computed LBM respectively, for the OVER and NORM subgroups. These allometric equations have the general form** $\boldsymbol{LVM}\boldsymbol{=}{\boldsymbol{a}\left( \boldsymbol{body} \boldsymbol{size} \right)}^{\boldsymbol{b}}$**, where a and b are the coefficient and exponent of the allometric equation, respectively.**

|  | **a** | **b** | **R^2^** |
| --- | --- | --- | --- |
| **LVM estimated from height based on** |  |  |  |
| equation for OVER | 33.66 | 2.87 | 0.78 |
| equation for NORM | 32.68 | 2.78 | 0.71 |
| **LVM estimated from BSA based on** |  |  |  |
| equation for OVER | 61.41 | 1.54 | 0.80 |
| equation for NORM | 70.92 | 1.45 | 0.79 |
| **LVM estimated from computed LBM based on** |  |  |  |
| equation for OVER | 4.47 | 0.91 | 0.79 |
| equation for NORM | 4.28 | 0,93 | 0.79 |
